# Supplementary material for: Learning curve and surgical outcome of robotic assisted colorectal surgery with ERAS program
Source: Sci Rep. 2022 Nov 29;12:20566. doi: 10.1038/s41598-022-24665-w (PMC9709162; doi:10.1038/s41598-022-24665-w)
Supplement: Supplementary file 1 — Supplementary Information 1. [file 41598_2022_24665_MOESM1_ESM.zip › LSC Raw data-Ñ[▒K/055/OP N_ñw╝╨░O▒Kñσ.pdf]

W95 / W95

臺 中 榮 民 總 醫 院

手術室護理記錄

[redacted]

基本資料

- 室別： 開刀房-06室，手術科別： CRS，手術類別： 常規，日期： 2019/12/12
- 進入時間： 1210 ； 麻醉開始時間： 1215 ； Time Out時間： 1305 ； 劃刀時間： 1306 ； 手術結束時間： 1700 送出時間： 1720 ；
- 麻醉方式： GE

  診斷：Malignant tumor of rectum

  手術名稱：1.Low anterior resection +/- pelvic LN dissection2.Laparoscopy3.4.

  手術局部用藥：NIL

  術前預防性抗生素：
  - 抗生素名稱一： Veterin 1GM IVD/由麻醉護理師給予
  - 時間： 1305
  - 上次簽章: 李郁萱

  ，追加預防性抗生素：
- 手術醫師： 蔣鋒帆，許家榮，黃俋霖，蔡濬樟
- 麻醉醫師： VS 王賜洲
-
  - 刷手護士A ： 李郁萱，時間： 1210，接替刷手一： 陳家瑋，時間： 1230，接替刷手二： 陳秋誼，時間： 1500
  - 流動護士A ： 呂昭蓉，時間： 1210，接替流動一： 李郁萱，時間： 1300

皮膚狀況

A-V Shunt： 無

術前： 完整

記錄者： 上次簽章: 呂昭蓉

術後： 完整

記錄者： 上次簽章: 李郁萱

手術準備

手術臥式 單一手術臥式

-
  - 手術臥式： 截石臥式
  - 使用附件： 溫毯 38 ℃，頭圈
  - 皮膚準備： 10%Aq Betadine，Hibiscrub，其它： 2%chlorhexidine
  - 電燒： 使用電燒: 電極片位置： 其它： 電燒迴路板，記錄者： 上次簽章: 呂昭蓉

止血帶T： 無

植入物

- 植入物名稱/號碼/數量/部位：ETHICON ENDO GIA60-4.1X2/ Colon

，記錄者： 上次簽章: 李郁萱

標本送檢

- 細菌培養 無
- 細胞學 無
- 冰凍切片 無
- 病理標本

  組織名稱一：IMA LN

  ，

  組織名稱二：IMA root

  ，

  組織名稱三：DISTAL CUT END

  ，更多組織名稱
  - 組織名稱四：RECTUM

- 截肢 無

標本交付 標本： 3 袋，標本： 0 盒，標本： 1 瓶，細胞學： 0 管，Culture Tube： 0 管，被交付醫師： CR 黃俋霖，

- 記錄者： 上次簽章: 李郁萱
- 時間： 1645

傷口術後數料

傷口數料： 傷口數料： 無

- 請於圖上標示部位：

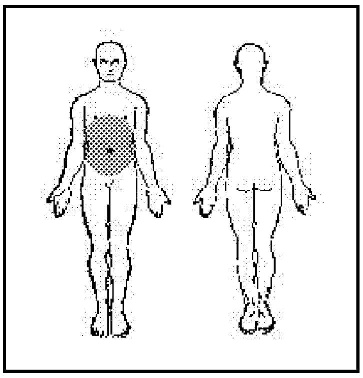

- 傷口數料： SOFT CLOTH

導管

導管	天數	導管資訊	插入日期	插入時間	更換日期	拔除日期	拔除時間	拔除原因	預計拔除/更換	預計拔除/更換日期
Penrose Drain(1)	1	號碼:12 mm.; 部位:骨盆腔;	2019/12/12	1610						
Foley(1)	1	號碼:16 Fr.; 部位:膀胱;	2019/12/12	1255						
A-line (1)	1	號碼:20 G; 部位:右手;	2019/12/12	1240						
Sump NG Tube(1)	1	號碼:16 Fr.; 深度:55cm; 部位:胃;	2019/12/12	1230						

用物計數

- 手術中出血量： MINIMAL c.c.
- 手術中尿量： 100 c.c.
-
    - 其它： 無

用物計數： 多切口同時縫合

- 1.器械： 正確，2.縫針： 正確，3.刀片： 正確，4.紗布： 正確，5.腹部墊： 未使用，6.紗球： 未使用，7.PEANUTS ： 未使用，8.紗條： 未使用，9.絲帶： 未使用，10.針頭： 未使用，11.Vessel loop： 未使用，12.Boots： 未使用，13.Jelco： 未使用，14.棉條： 未使用，
    - 第一次：
        - 刷手護士 ： 陳秋誼
        - 流動護士： 上次簽章: 李郁萱
        - 時間： 1640

  ,
    - 第二次：
        - 刷手護士 ： 陳秋誼
        - 流動護士： 上次簽章: 李郁萱
        - 時間： 1645

  ,
    - 第三次：
        - 刷手護士 ： 陳秋誼
        - 流動護士： 上次簽章: 李郁萱
        - 時間： 1650

備註

備註： OLYMPUS SCOPY NO.2

- 記錄者： 李郁萱 上次簽章: 李郁萱
- 時間： 1720

填寫護理人員 李郁萱

正式摘要

		W95 / W95			

doctorRank:B
